# Identifying genetic relationships among tarsier populations in the islands of Bunaken National Park and mainland Sulawesi

**DOI:** 10.1371/journal.pone.0230014

**Published:** 2020-03-26

**Authors:** Thalita Christiani Pingkan Sumampow, Myron Shekelle, Paul Beier, Faith M. Walker, Crystal M. Hepp

**Affiliations:** 1 School of Forestry, Northern Arizona University, Flagstaff, AZ, United States of America; 2 Western Washington University, Bellingham, WA, United States of America; 3 Pathogen and Microbiome Institute, Northern Arizona University, Flagstaff, AZ, United States of America; 4 School of Informatics, Computing, and Cyber Systems, Northern Arizona University, Flagstaff, AZ, United States of America; Sichuan University, CHINA

## Abstract

Eastern tarsiers (*Tarsius tarsier* complex) are small nocturnal primates endemic to Sulawesi Island and small adjacent islands of Indonesia. In 2004, the hybrid biogeography hypothesis predicted this species complex might contain 16 or more taxa, each corresponding to a region of endemism, based on: 1) geological evidence of the development of the archipelago, 2) biological evidence in the form of concordant distributions of monkeys and toads, and 3) the distribution of tarsier acoustic groups. Since then, 11 tarsier species have been recognized, potentially leaving more to be described. Efforts to identify these cryptic species are urgently needed so that habitat conversion, pet trade, and cultural activities will not render some species extinct before they are recognized. We gathered data to test the hypothesis of cryptic tarsier species on three volcanic islands in Bunaken National Park, North Sulawesi, namely Bunaken, Manadotua, and Mantehage, during May-August 2018. We sequenced individuals at 5 nuclear genes (ABCA1, ADORA3, AXIN1, RAG, and TTR) and made comparisons to existing genotypes at 14 mainland sites. Bayesian phylogenetic analyses revealed that island populations are genetically identical in all 5 genes, and formed a clade separated from the mainland ones. The eastern tarsiers first diverged from the western tarsiers approximately 2.5 MYA. The three island populations diverged from mainland tarsiers approximately 2,000–150,000 YA, due to either human activities or natural rafting. This study provides information for tarsier conservation, advances the understanding of biogeography of Sulawesi, and contributes to Indonesian awareness of biodiversity. Further quantitative genetics research on tarsiers, especially the island populations, will offer significant insights to establish more efficient and strategic tarsier conservation actions.

## Introduction

The Catalogue of Life lists more than 1.8 million eukaryotic species [1], leaving ~25–60% of the estimated total number of all species on Earth undescribed [2–4]. One obstacle to cataloging biological diversity is the presence cryptic species—two or more genetically distinct species sharing similar morphological traits and therefore classified as a single species. A shortage of taxonomists and funding limits response to the problem [5–7].

In light of increasing threats to biodiversity [8], accurate species identification is critical to setting conservation priorities and strategies [9], understanding species diversification and other biological processes [4, 10], and clear communication among researchers and non-researchers [4]. Particularly, the discovery and accurate description of cryptic species can improve estimates of species distributions, endemism, and conservation priorities [11–14]. Identification of cryptic species could also improve information regarding medically and economically important species [6] as well as reduce risk of pathogen spread [15–16] and errors in use of traditional medicines [17–18].

Eastern tarsiers (*Tarsius* spp.) are endemic to Sulawesi Island and small adjacent islands of Indonesia. They were classified as a single species, *Tarsius tarsier* (= *spectrum*), as recently as 1984 [19], but observations of geographically-structured variation of vocalizations among populations [20] provoked additional taxonomic research e.g., [13, 21–26] that led to the resurrection of several taxa, namely *T*. *pumilus* [27], *T*. *sangirensis* [28] in [24], *T*. *dentatus* (= *dianae*) [21, 29], *T*. *pelengensis* [30], and the recognition of several new taxa, *T*. *lariang*, [31], *T*. *tumpara*, [32], *T*. *wallacei*, [33], *T*. *spectrumgurskyae*, and *T*. *supriatnai* [25]).

The identification of cryptic tarsier species has followed a process familiar in other nocturnal primates [34]: a combination of three lines of evidence are employed, typically in a sequence starting with regionally distinct vocalizations [13, 20, 25, 35, 36] followed by genetic evidence of differences among acoustic groups [33, 37–39], and ending with identification of subtle morphological indicators such as skin color, tail length, and furriness of tail tufts [12, 26, 31, 40]. This combination has been more informative than morphological approaches alone [19, 27]. Studies on genetic patterns and distinctive vocalizations e.g., [36, 37, 39, 41] have recently led to the recognition of two new tarsier species, *T*. *supriatnai* and *T*. *spectrumgurskyae* [25], bringing the total to 11 species. The hybrid biogeographic hypothesis [24] observes that 16 bioacoustically distinct populations of eastern tarsiers correspond to the intersection of those geologically distinct regions of Sulawesi identified by Hall [42] and those biologically distinct regions that show concordant distributions of monkeys and toads (plus those offshore islands that have tarsiers, but which lack monkeys), and thus likely represent 16 distinct species. As 11 species have been recognized in the predicted regions, five potential species still left to be described, and others may yet be undiscovered.

Because tarsiers are threatened by habitat loss, small population sizes, wildlife trade, and local exploitation, Shekelle et al. [32] concluded that “some primate species in Sulawesi may go extinct before they have even been identified.” Consisting of cryptic species, eastern tarsiers are difficult to identify solely using morphology evidence. Therefore, rapid identification efforts, such as collecting genetic evidence, are urgently needed to prevent the animals from early extinction.

**Fig 1:**
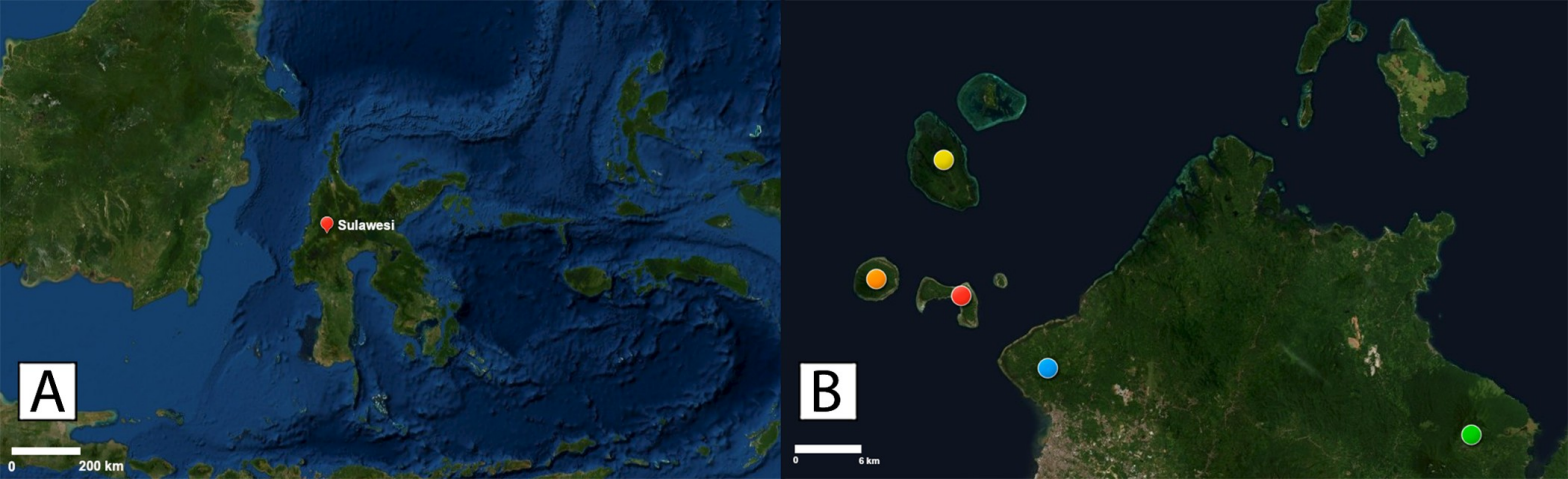
The maps of Sulawesi and parts of North Sulawesi. A) Sulawesi, the fourth largest island in Indonesia, hosts many endemic animals. The “hybrid biogeography hypothesis” (Shekelle and Leksono, 2004) suggests that there are at least 16 subregions of tarsier endemism throughout the island. B) Grand forest park Gunung Tumpa (blue circle), Duasudara Nature Reserve (green circle), and three islands in TNB: Bunaken (red circle), Manadotua (orange circle), Mantehage (yellow circle).

We collected genetic data on tarsier populations on three islands of Bunaken National Park (TNB) in North Sulawesi, namely Bunaken, Manadotua, and Mantehage, that do not appear in previously published records of tarsier distribution, but which have logically been regarded as conspecific with tarsiers on the eastern northern peninsula of mainland Sulawesi near Duasudara, Bitung (*T*. *spectrumgurskyae*, Fig 1). However, preliminary comparisons of their vocal patterns compared to the Gunung Tumpa population (Fig 1) suggest they may be unique species [43]. Furthermore, the three islands of TNB qualify as biogeographically unique [24, 29, 44]. Genetic evidence is entirely lacking on these islands and might yield persuasive evidence.

We analyzed the genetic data to determine the genetic uniqueness of tarsiers on each island, and their phylogenetic relationships with Sulawesi tarsiers. Although some local people believe the Bunaken population may have been introduced by humans < 20 years ago, the populations on the other two islands are thought to have been established by natural dispersal events. Our goal was to help test the predictions of hybrid biogeographic hypothesis of Shekelle and Leksono [24], that each of these populations was a distinct species. In pursuing this goal, we also hope to contribute to conservation of tarsiers and the forest on which they depend, and the other species that also occupy those forests–likely including other endemic and un-named species [25].

## Methods

### Study area

**Fig 2:**
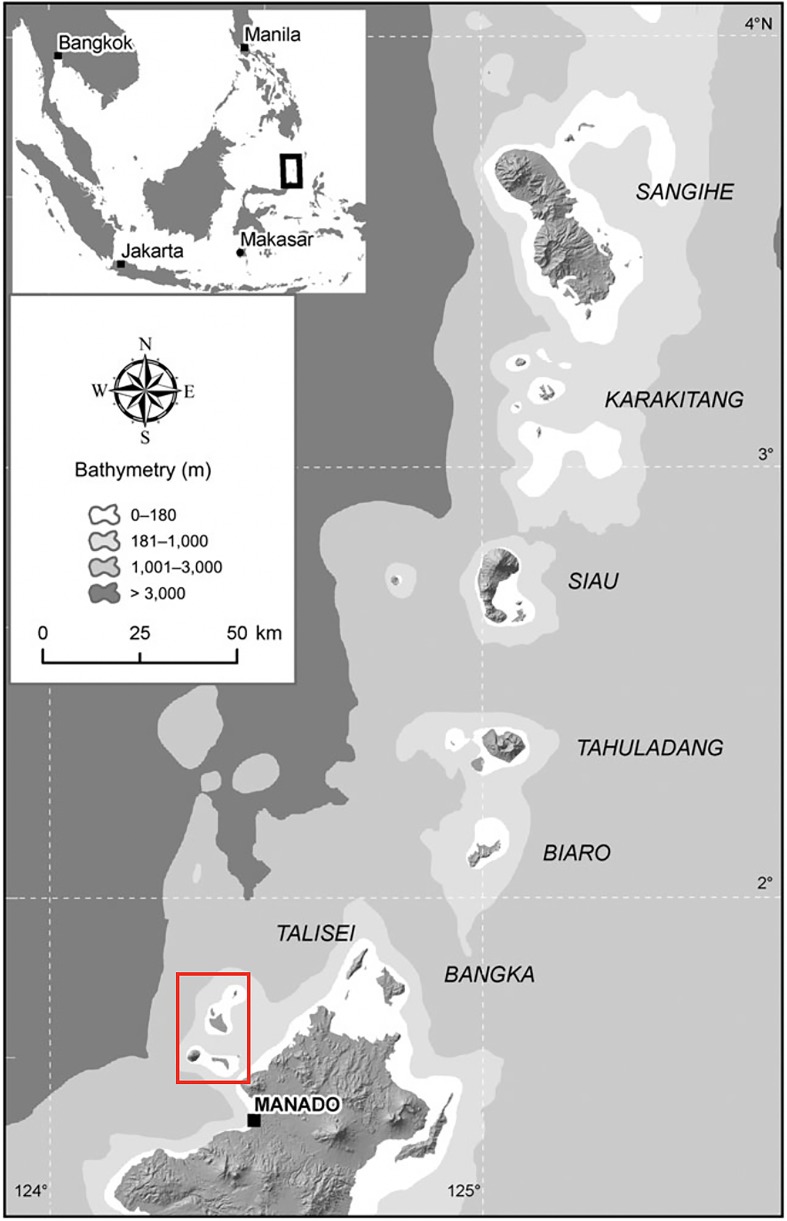
Bathymetry map of northern Sulawesi archipelago (from Shekelle and Salim, 2009). The islands of Bunaken National Park (TNB) are in red box.

Field work was conducted during May–August 2018 on three islands in TNB, North Sulawesi: Bunaken (size = ~8 km^2^), Manadotua (10 km^2^), and Mantehage (15 km^2^) (Fig 1). These volcanic islands are believed to have arisen independently, and ocean waters probably preclude regular gene flow between islands (Fig 2) [44]. Each of these islands qualifies as a potentially distinct biogeographic region in Shekelle and Leksono’s [24] hybrid biogeography hypothesis, suggesting that each site may harbor a distinct tarsier species.

### Tissue collection and genetic analysis

**Fig 3:**
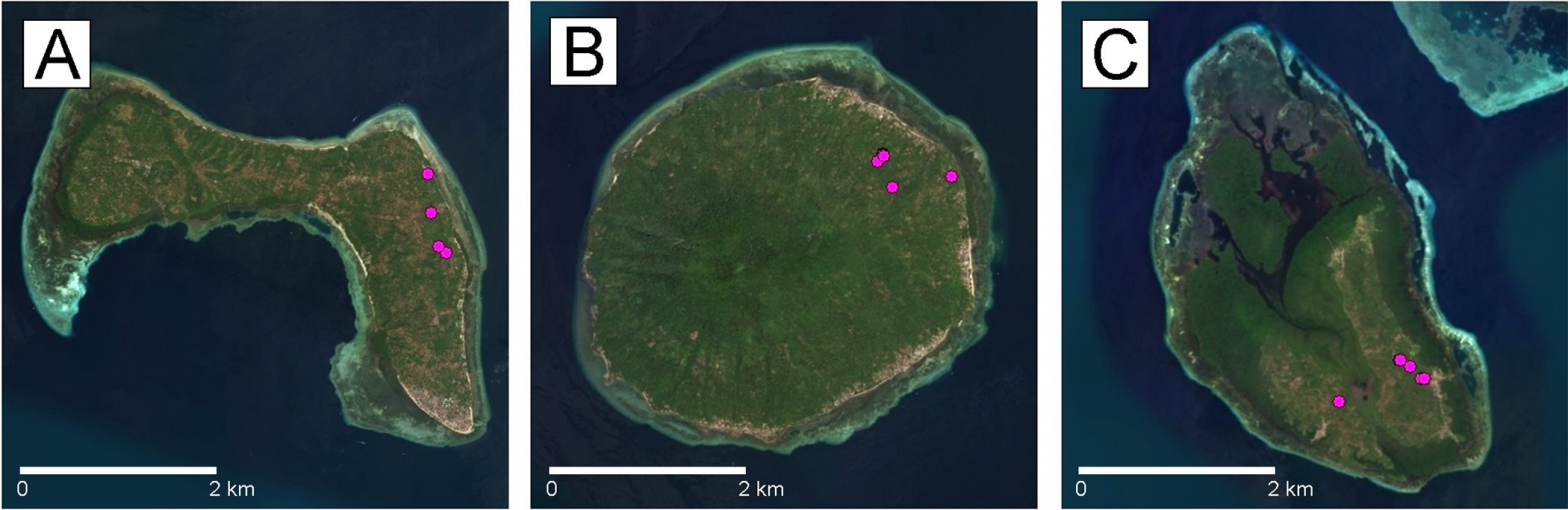
The approximate locations of tarsier sleeping sites on the study sites. A) Bunaken, B) Manadotua, and C) Mantehage.

Prior to data collection, we spent 3–5 days on each island to locate sleeping sites by following tarsiers’ scent marks and duet calls. Time constraints hindered from exploring the entire island. To minimize the effect of sampling individuals that are more closely related than a random subset of animals, we tried to select widely-spaced sites, but our samples were clustered with respect to the total island area (Fig 3). Some other parts of the islands have been altered into plantation area which are not a suitable habitat for tarsier. Hence, the low number of individuals. We documented the GPS coordinates of all sleeping sites, plants within approximately 10 m of any sleeping sites, and the plant used for the sleeping site. Use of wild tarsiers and the animal-handling protocol in this research was approved by the Institutional Animal Care and Use Committee at Northern Arizona University (protocol number: 18–001).

To collect DNA samples, we captured tarsiers by setting up mist nets near sleeping sites. Following capture, individuals were placed in a cloth bag and carried to a nearby processing station. We sampled saliva by buccal swab (Whatman sterile omni swab) and took a small ear pinna biopsy (2 mm) using Miltex Disposable Biopsy Punch. We used a flashlight to find and avoid veins and applied silver nitrate, iodine, and/or 70% alcohol solution onto the tarsiers’ ear to stop bleeding and prevent infection. The tissue samples were stored and preserved separately in a 1.5 ml microtube with RNAlater solution. After we measured and photographed the tarsier, we immediately released it alive. Prior to the release tarsiers were held for approximately 15–20 minutes. The animal sampling and handling is acknowledged by The Ministry of Environment and Forestry Republic of Indonesia (S.616/KKH/SDG/KSA.2/5/2018).

All genetic samples were brought to the biotechnology lab at *Universitas Sam Ratulangi*, North Sulawesi for DNA extraction (DNeasy Blood and Tissue Kit, Qiagen) and amplification for the five nuclear genes using previously developed primers [39]. PCR products were sent to 1^st^ BASE Malaysia for the sequencing of 5 nuclear genes of “phylogenomic toolkit” (ABCA1, ADORA3, AXIN1, RAG1, TTR) [45] using the Sanger method. We trimmed the ends of the sequences in Geneious 10.2.6 [46] and aligned them using MEGA7 [47]. Concatenation of genes was accomplished in SequenceMatrix [48].

**Fig 4:**
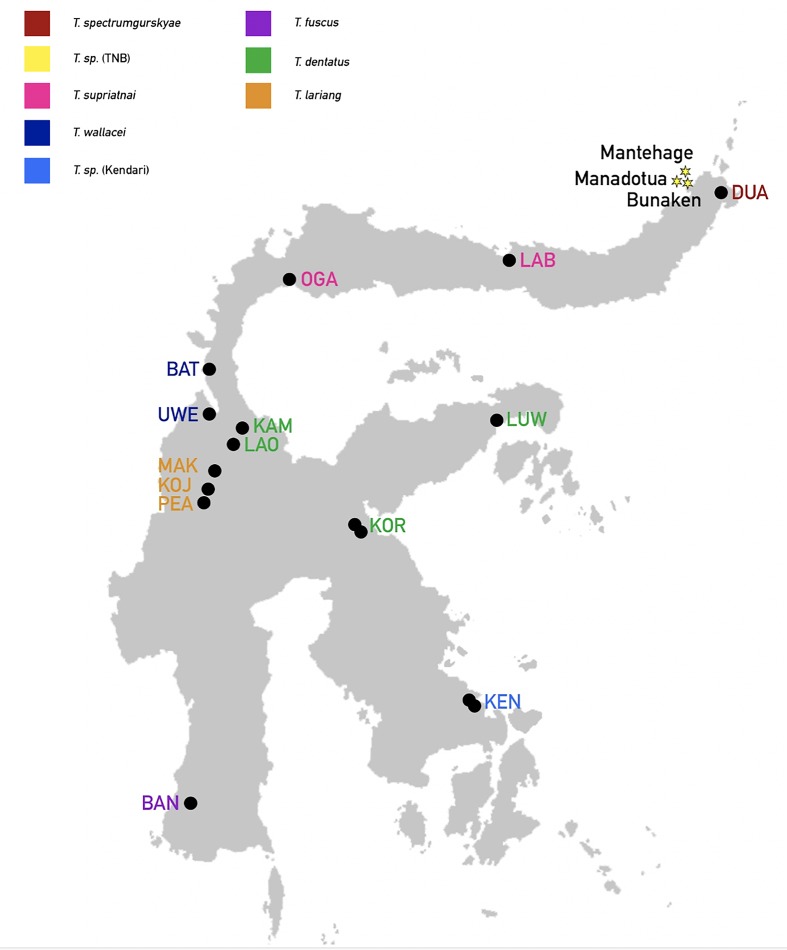
Origins of DNA sample from 17 populations of eastern tarsier. Stars: this study (red: Bunaken; orange: Manadotua; yellow: Mantehage; see Fig 1). Black dots: Driller et al. (2015).

To construct phylogenetic trees, we obtained sequences of the same 5 nuclear genes for western tarsiers (one individual), Philippine tarsiers (one individual), and 2 individuals from each of 14 mainland populations of eastern tarsier (Fig 4) from Driller et al. [39].

We used BEAST v1.10.4 to estimate the divergence times based on five nuclear genes of the primates phylogenetic markers [45]. We used 15 interior node calibration times described in Driller et al. [39]. Because these are secondary timing estimates determined in previous molecular clock analyses, we used a normal distribution as a calibration prior around the mean within a single standard deviation [49]. Prior to the Bayesian analysis, we determined the best substitution models for each gene in MEGA7, which was in agreement with Driller et al. [39] (Table 1). We estimated the divergence times under strict and uncorrelated lognormal relaxed clocks using a gamma distribution as prior on branch-specific substitution rates. We ran four independent BEAST analyses for each of the clock models for 10x10^7^ generations, sampling every 10,000^th^ generation. We analyzed the trace files in Tracer v1.7.1 to confirm convergence within and among Markov chains [50]. We then resampled the combined trace files every 40,000 generations in LogCombiner v1.10.4 to produce 9,000 trees. The maximum clade credibility tree was identified using TreeAnotator v1.10.4, and associated statistics were summarized for all nodes. Trees were visualized and refined in FigTree v1.4.4 [51].

**Table 1:** Proposed best-fit substitution models for each genetic marker based on Akaike’s information criterion corrected for small sample sizes (AICc) using MEGA7.

Locus	Species tree model
ABCA1	HKY
ADORA3	HKY+G
AXIN1	HKY+G
RAG1	TN93
TTR	T92+I

(HKY: Hasegawa, Kishino and Yano model (Hasegawa et al., 1985), TN93: Tamura-Nei two parameter model (Tamura and Nei, 1993); T92: Tamura 3-parameter (Tamura, 1992); I: Invariable sites model; G: Discrete Gamma model).

## Results

We found a total of 17 sleeping sites (seven on Manadotua, four on Bunaken, six on Mantehage) during our field efforts. These sites were surrounded by *Arenga* (palms), *Bambusa* (bamboos), *Cocos* (coconut trees), *Ficus* (benjamin fig trees), lianas, shrubs, and crops (cassava, maize, and banana) (Table 2). We collected genetic material from 18 individuals (three from Manadotua, four from Bunaken, and eleven from Mantehage) at nine tarsier sleeping sites (Fig 3).

**Table 2:** Vegetation around tarsier sleeping sites on Bunaken, Manadotua, and Mantehage islands.

Sleeping site	Vegetation						
	*Bambusa*	*Arenga*	*Ficus*	*Cocos*	crops	lianas	shrubs
BS1	**S**						P
BS2	**S**				P		P
BS3	**S**					P	P
BS4*	**S**			P		P	P
DS1	P	**S**		P	P		
DS2	P	**S**		P			
DS3	**S**	P		P			
DS4*	P	**S**		P			
DS5*	**S**			P	P	P	
DS6*	**S**	P		P	P		
DS7*		**S**		P	P		
HS1	**S**			P			
HS2		**S**	P	P			P
HS3		P	P	P	**S**		
HS4	**S**	P		P	P	P	P
HS5*		P	**S**	P			P
HS6*	P	P		P	P	P	
**Frequency**	0.76	0.65	0.18	0.82	0.47	0.29	0.41

S = sleeping site plant. P = plant presents within approx. 10 m of sleeping site.

* = efforts to capture tarsiers were unsuccessful.

**Fig 5:**
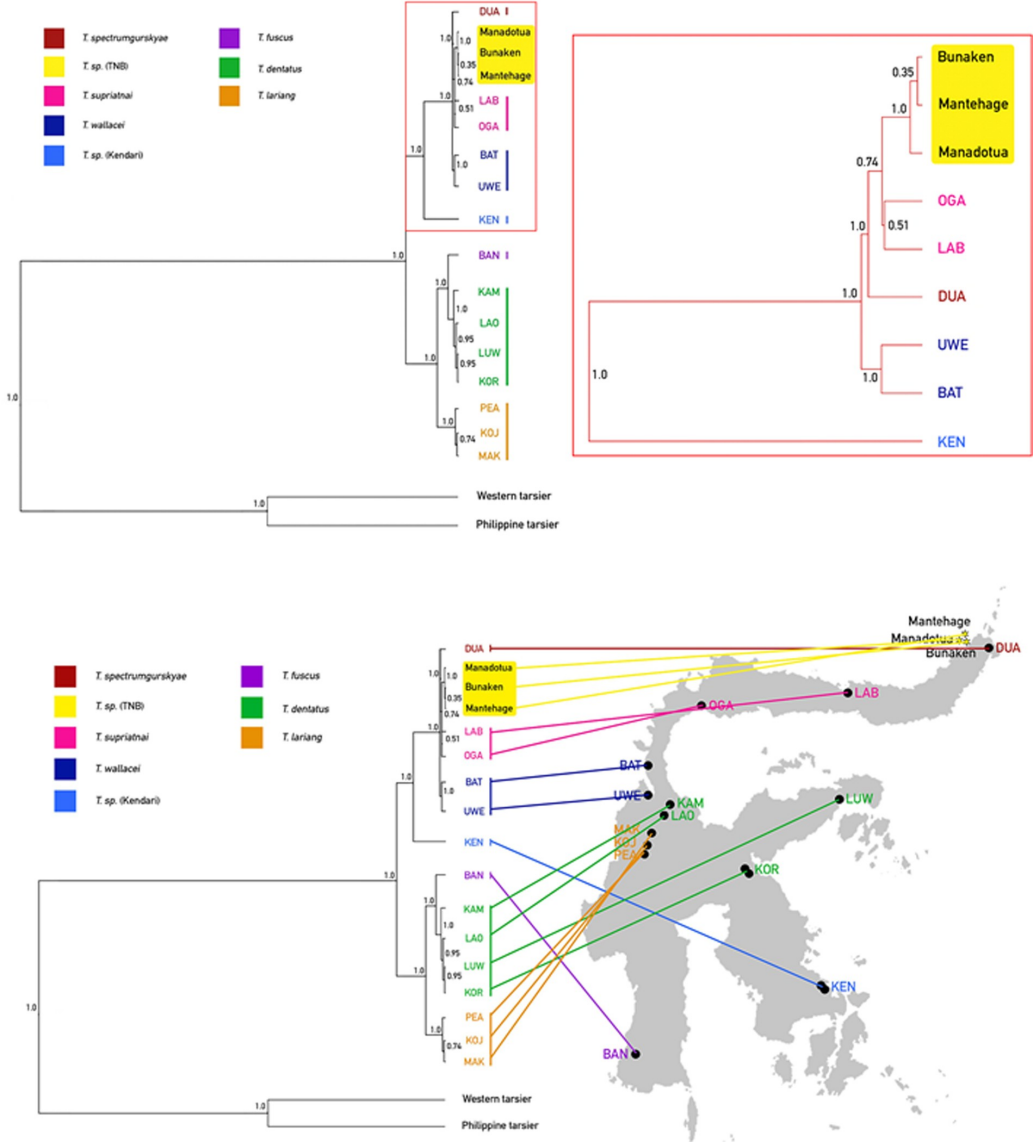
Multilocus Bayesian species tree. Northern and southeastern Sulawesi populations are highlighted in red. (A) full tree; (B) distribution map of 17 populations of eastern tarsier; (C) detail view of the red box in the upper panel.

Multilocus Bayesian species tree inference revealed two well-supported evolutionary lineages of eastern tarsier (Fig 5). The southeastern population of *Tarsius* sp. KEN represents is the first to diverge within lineage 1 and is a sister group to all other northern populations. Among those, the north-central *T*. *wallacei* (BAT and UWE) is distinct from the northern DUA, LAB, OGA, Manadotua, Bunaken, and Mantehage populations, and form a monophyletic group. Identical sequences of *Tarsius* sp. of TNB (Manadotua, Bunaken, Mantehage) (see S1 Table for the SNPs between each of the population) form a single clade and is a sister group to two northern mainland populations (LAB and OGA, pp = 0.51). Within lineage 2, the central *T*. *lariang* (PEA, KOJ, and MAK) is a sister group to *T*. *fuscus* and *T*. *dentatus* (pp = 1.0). The southern population of *T*. *fuscus* BAN is placed as a sister group to *T*. *dentatus* (pp = 1.0). In general, eastern tarsier form a well-supported single clade that is separated from western and Philippines tarsier (pp = 1.0).

**Fig 6:**
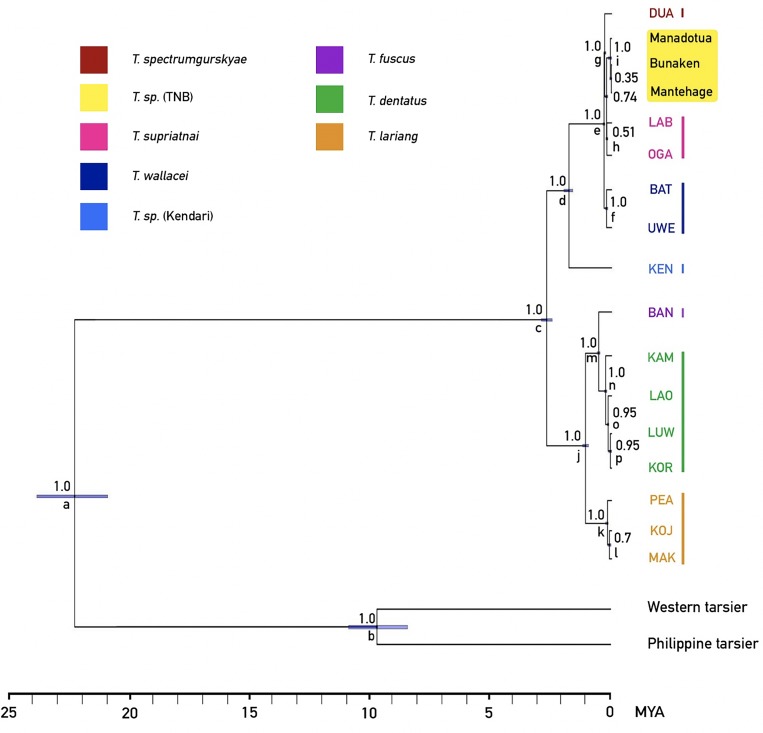
Time-calibrated multilocus Bayesian tree. Numbers above nodes are posterior probability (pp) and 95% confidence intervals are indicated with purple bars. Lowercase letters indicate nodes’ names (see Table 3). Study populations are yellow highlighted.

Based on our Bayesian chronogram (Fig 6), the eastern lineage split from other crown tarsiers between late Oligocene and early Miocene (median node age calibration: 22.27 MYA, 95% confidence intervals ranging from 20.96–23.62 MYA). Western and Philippine tarsiers diverged about 10 MYA (median node age calibration: 9.78 MYA, 95% confidence interval ranging from 8.87–10.7 MYA). Eastern tarsier split into 2 lineages around 2.5 MYA (median node age calibration: 2.54 MYA, 95% confidence interval ranging from 2.32–2.76 MYA). The speciation events of both lineages occurred during Pleistocene and early Holocene (1.6–0.1 MYA). The northern island populations (Manadotua, Bunaken, Mantehage) diverged from mainland populations during late Pleistocene and early Holocene (median node age calibration: ~69,000 YA, 95% confidence interval ranging from 150,000–2,000 YA) (see Table 3).

**Table 3:** Divergence time estimates based on a time-calibrated multilocus Bayesian tree (see Fig 5).

Node	Node label	Median	95% highest posterior density (HPD)		
		Node age (MYA)	Lower	Upper	pp*
Tarsius	a	22.27	20.96	23.62	1.0
	b	9.78	8.87	10.7	1.0
Lineage 1	c	2.54	2.32	2.76	1.0
	d	1.67	1.5	1.85	1.0
	e	0.3	0.27	0.34	1.0
	f	0.2	0.16	0.25	1.0
	g	0.27	0.23	0.31	1.0
	h	0.19	0.15	0.23	0.51
	i	0.06	0.002	0.15	1.0
Lineage 2	j	1.02	0.9	1.13	1.0
	k	0.17	0.14	0.24	1.0
	l	0.16	0.12	0.19	0.74
	m	0.51	0.46	0.57	1.0
	n	0.51	0.46	0.57	1.0
	o	0.24	0.19	0.29	0.95
	p	0.14	0.11	0.18	0.95

* = posterior probability.

## Discussion

### Sleeping sites

Mantehage is a low flat island composed principally of mangrove forest, with some areas cleared for agricultural use. Tarsiers are likely to be common in mangrove [44, 52] but because of the difficulty of surveying them in mangrove, we selected capture sites in or near habitats modified for human use. The forested areas were mostly altered to coconut, palm (locally called *seho*), and crop plantations (agroforestry). Almost all native forest on Bunaken, similarly low and flat like Mantahage, have been converted to palm plantations, and tourist cottages, resorts, and shops. Manadotua is a stratovolcano with a steep cone and very little flat terrain. Forests are protected by the steepness of the slope, but human alterations are nevertheless common.

Coconut trees occurred near 82% of sleeping sites, consistent with the dominance of coconut plantations on all three islands (personal observation). Mainland eastern tarsier were commonly observed to sleep in a fig tree that has spacious dark hollow trunk [53]. However, the animals were also documented to occur in various types of vegetation [20]. Our data are consistent with previous studies that show tarsier’s ability to be present in diverse environments [54–57].

### Divergence of eastern tarsiers

Our multilocus Bayesian species tree (Fig 6) is congruent with Driller et al.’s [39], except for the BAN population and the addition of the TNB populations. The speciation of eastern tarsier populations began ~2.5 MYA during the glacial maximum [58] when the genus *Tarsius* split into 2 lineages: northern to southeastern and central to southern populations. The split of northern (BAT, UWE, OGA, LAB, DUA, Manadotua, Bunaken, Mantehage) and southeastern (KEN) populations in early Pleistocene (~1.67 MYA) might be caused by the strike-slip faulting and formation of Malili Lake in central Sulawesi [59]. The drop of sea-level during the Pleistocene ice age might have given northern population access to migrate up to the most north tip of the peninsula [39]. Some more recent interglacial sea-level rises in late Pleistocene may have further subdivided populations in northern Sulawesi (~300–150 MYA) [60–61], but the short-lived water barriers may have been insufficient for complete speciation. Several alternative causes are 1) smaller distribution range due to natural events, such as volcano formations and eruptions [62], or anthropogenic (hominin) events [63], 2) smaller population size, and 3) assortative mating. The first two mechanisms might result in divergence through interspecific hybridization [64–65] while assortative mating could have promoted genetic heterogeneity among recently evolved northern tarsier populations [39].

Mehta [62] provides the first record of tarsier populations on Mantehage, Bunaken, and Manadotua. These populations have been considered conspecific with the mainland *T*. *spectrumgurskyae* (*T*. *sp* DUA) (Tasirin, *pers*. *comm*., 2019). However, the hybrid biogeographic hypothesis [24] and Schuler’s recent vocalization data [43] suggest that these populations are distinct from the ones on the mainland. Furthermore, because the islands arose independently as seafloor volcanoes [42] each island could plausibly have a distinct tarsier species. However, our finding that the sequences of the phylogenetic markers (see previous section) of three island populations are all identical (S1 Table), suggests that these tarsier populations are a single subspecies or species that arrived within the last 150,000 years (median node age calibration: ~69,000 YA, 95% confidence interval: 150,000–2,000 YA). A single clade for the TNB populations is consistent with Schuler’s finding of the vocal patterns on Manadotua, Mantehage, and Bunaken (Schuler found no tarsiers on Nain and Siladen) that are distinct from that of mainland population (Mt. Tumpa).

Based on our analysis of divergence times estimates (Fig 6), the migration and speciation of TNB populations occurred 2,000 to 150,000 YA, the most recent among other northern Sulawesi populations. Our phylogenetic analysis indicates that the TNB populations are most closely related to OGA and LAB (*T*. *supriatnai*). Because vicariance is not an option owing to the independent formation of the islands [42, 62, 66] and lack of evidence for any landbridge between each of the islands and to the mainland, the migration of tarsiers of TNB was either by natural (oceanic) or anthropogenic dispersal. *Homo sapiens* colonized Sulawesi 40,000 to 60,000 YA [63, 67]. Furthermore, some recoveries of remains of ancient megafauna, such as *Stegodon*, that are associated with stone artefacts in South Sulawesi dated between 118,000–194,000 YA, indicate much earlier occupation of hominins (possibly *H*. *erectus* or *H*. *floresiensis*) in Sulawesi [63]. In addition, stone tools and butchered *Rhinoceros philippinensis* in northern Luzon, Philippine dated to ~700,000 YA suggest an even older colonization of ancient hominins (presumably *H*. *luzonensis* [68]) in Southeast Asia [69]. Thus, tarsiers could have migrated to TNB by either ancient or modern human action, or natural rafting. Because Bunaken is closest to the mainland (3.5 km), it may have been the first island colonized (Figs 1 and 2), perhaps by rafting of logs from lowland and coastal plants of North Sulawesi’s paleo-vegetation [60] in ocean current [70] or a tsunami [71]. Rafting between the islands is possible for tarsiers because the islands are close together (approximately 2–8 km between each island) (Figs 1 and 2). Bathymetry (Fig 2) suggests the islands were not connected during glacial maxima.

## Conclusions

Based on the results, we conclude that:

1. Based on the five phylogenetic markers (ABCA1, ADORA3, AXIN1, RAG1, TTR), tarsier populations in Bunaken National Park (TNB) form one genetic clade distinct from tarsiers of mainland Sulawesi.
2. Additional genetic studies of tarsiers on TNB and other parts of mainland North Sulawesi (such as Mt Tumpa–Fig 1) are needed to elucidate gene flow among the 3 islands and identify the most closely related mainland populations. These efforts should include additional nuclear genes, mitochondrial loci and perhaps whole genome sequencing, and population genetics approach using microsatellites or the other faster evolving marker to analyze the genetic variation within and among population quantitatively.
3. Additional recording of vocalizations and morphology are needed to confirm the taxonomy of TNB tarsier populations.
4. Population estimates and habitat preferences are needed to develop conservation management plans.

### Management implications

Bunaken National Park (TNB) currently protects marine areas with very limited terrestrial sites [72, 73]. We hope the discovery of a genetically distinct population of tarsiers in TNB will strengthen the government’s plan to review the TNB zonation and expand protection to larger terrestrial areas. Our finding that tarsiers in TNB (like those on the mainland) tolerate moderate amounts of disturbance suggests that such protection might be compatible with some human activities as long as sleeping sites are well protected, including areas where vegetation can create new sleeping sites as old sites are lost to plant age, typhoons, or tsunamis.

The finding of these novel populations may raise the local awareness of natural resource conservation. This could also be an opportunity for academia and local government to educate local communities about community-based conservation efforts, like one that has been done in Tangkoko-Duasudara Nature Reserve, and the tarsier sanctuary in Bohol, Philippines. Such an approach would not only benefit the environment but also foster economic growth through well-managed ecotourism.

This study will also help the government develop both regional and federal laws on wildlife conservation, more specifically in the revision of the Ministry’s lists of endangered and protected animals. Furthermore, we encourage the provincial government to have stronger law enforcement on illegal hunting and pet trade, as well as land-use conversion. Lastly, the project provides the IUCN new, reliable information on tarsiers that will result in more accurate conservation status and species assessment.

## Supporting information

S1 Table Single nucleotide polymorphisms (SNPs) shared by pairs of tarsier populations. The gaps separated individuals from the next closest species. The numbers are in basepairs (bp). The total length of 5 nuclear genes sequenced is 3,374 bp.
(PDF)
