# Supplementary material for: Identifying genetic relationships among tarsier populations in the islands of Bunaken National Park and mainland Sulawesi
Source: PLoS One. 2020 Mar 26;15(3):e0230014. doi: 10.1371/journal.pone.0230014 (PMC7098592; doi:10.1371/journal.pone.0230014)
Supplement: S1 Table — The gaps separated individuals from the next closest species. The numbers are in basepairs (bp). The total length of 5 nuclear genes sequenced is 3,374 bp. (PDF) [file pone.0230014.s001.pdf]

Supplementary Table 1. Single-nucleotide polymorphisms (SNPs) shared by pairs of tarsier populations. The gaps separated individuals from the next closest species. The numbers are in basepairs (bp). The total length of 5 nuclear genes sequenced is 3,374 bp.

	Bunaken (5 gaps)	Manadotua (5 gaps)	Mantehage (5 gaps)	DUA (3 gaps)	LAB (5 gaps)	OGA (5 gaps)	BAT (3 gaps)	UWE (3 gaps)	KEN (4 gaps)
Bunaken (5 gaps)		3,369 (100%)	3,369 (100%)	3,361 (99.7%)	3,365 (99.9%)	3,365 (99.9%)	3,359 (99.6%)	3,357 (99.6%)	3,355 (99.6%)
Manadotua (5 gaps)	3,369 (100%)		3,369 (100%)	3,361 (99.7%)	3,365 (99.9%)	3,365 (99.9%)	3,359 (99.6%)	3,357 (99.6%)	3,355 (99.6%)
Mantehage (5 gaps)	3,369 (100%)	3,369 (100%)		3,361 (99.7%)	3,365 (99.9%)	3,365 (99.9%)	3,359 (99.6%)	3,357 (99.6%)	3,355 (99.6%)
DUA (3 gaps)	3,361 (99.7%)	3,361 (99.7%)	3,361 (99.7%)		3,364 (99.8%)	3,362 (99.7%)	3,362 (99.7%)	3,360 (99.7%)	3,355 (99.5%)
LAB (5 gaps)	3,365 (99.9%)	3,365 (99.9%)	3,365 (99.9%)	3,364 (99.8%)		3,365 (99.9%)	3,360 (99.7%)	3,359 (99.6%)	3,357 (99.6%)
OGA (5 gaps)	3,365 (99.9%)	3,365 (99.9%)	3,365 (99.9%)	3,362 (99.7%)	3,365 (99.9%)		3,361 (99.7%)	3,358 (99.6%)	3,356 (99.6%)
BAT (3 gaps)	3,359 (99.6%)	3,359 (99.6%)	3,359 (99.6%)	3,362 (99.7%)	3,360 (99.7%)	3,361 (99.7%)		3,364 (99.8%)	3,354 (99.5%)
UWE (3 gaps)	3,357 (99.6%)	3,357 (99.6%)	3,357 (99.6%)	3,360 (99.7%)	3,359 (99.6%)	3,358 (99.6%)	3,364 (99.8%)		3,357 (99.6%)
KEN (4 gaps)	3,355 (99.6%)	3,355 (99.6%)	3,355 (99.6%)	3,355 (99.6%)	3,357 (99.6%)	3,356 (99.6%)	3,354 (99.5%)	3,357 (99.6%)	
BAN (5 gaps)	3,352 (99.5%)	3,352 (99.5%)	3,352 (99.5%)	3,351 (99.4%)	3,355 (99.6%)	3,352 (99.5%)	3,350 (99.4%)	3,353 (99.5%)	3,353 (99.5%)
KAM (5 gaps)	3,352 (99.5%)	3,352 (99.5%)	3,352 (99.5%)	3,352 (99.4%)	3,354 (99.6%)	3,353 (99.5%)	3,351 (99.4%)	3,354 (99.5%)	3,354 (99.5%)
LAO (5 gaps)	3,350 (99.4%)	3,350 (99.4%)	3,350 (99.4%)	3,350 (99.4%)	3,352 (99.5%)	3,351 (99.5%)	3,349 (99.3%)	3,352 (99.4%)	3,352 (99.5%)

LUW (5 gaps)	3,353 (99.5%)	3,353 (99.5%)	3,353 (99.5%)	3,351 (99.4%)	3,354 (99.6%)	3,352 (99.5%)	3,350 (99.4%)	3,353 (99.5%)	3,353 (99.5%)
KOR (5 gaps)	3,353 (99.5%)	3,353 (99.5%)	3,353 (99.5%)	3,351 (99.4%)	3,354 (99.6%)	3,352 (99.5%)	3,350 (99.4%)	3,353 (99.5%)	3,353 (99.5%)
PEA (17 gaps)	3,338 (99.1%)	3,338 (99.1%)	3,338 (99.1%)	3,336 (99.0%)	3,339 (99.1%)	3,337 (99.1%)	3,335 (98.9%)	3,338 (99.0%)	3,338 (99.1%)
KOJ (17 gaps)	3,337 (99.1%)	3,337 (99.1%)	3,337 (99.1%)	3,335 (98.9%)	3,338 (99.1%)	3,336 (99.0%)	3,334 (98.9%)	3,337 (99.0%)	3,337 (99.0%)
MAK (17 gaps)	3,336 (99.0%)	3,336 (99.0%)	3,336 (99.0%)	3,334 (98.9%)	3,337 (99.1%)	3,335 (99.0%)	3,333 (98.9%)	3,336 (99.0%)	3,336 (99.0%)
Carlito (13 gaps)	3,194 (94.7%)	3,194 (94.7%)	3,194 (94.7%)	3,192 (94.6%)	3,193 (94.7%)	3,191 (94.7%)	3,192 (94.6%)	3,195 (94.7%)	3,192 (94.7%)
Cephalopachus (7 gaps)	3,221 (95.6%)	3,221 (95.6%)	3,221 (95.6%)	3,219 (95.4%)	3,220 (95.5%)	3,218 (95.5%)	3,219 (95.4%)	3,222 (95.5%)	3,219 (95.5%)

	BAN (5 gaps)	KAM (5 gaps)	LAO (5 gaps)	LUW (5 gaps)	KOR (5 gaps)	PEA (17 gaps)	KOJ (17 gaps)	MAK (17 gaps)	Carlito (13 gaps)	*Cephalopachus* (7 gaps)
Bunaken (5 gaps)	3,352 (99.5%)	3,352 (99.5%)	3,350 (99.4%)	3,353 (99.5%)	3,353 (99.5%)	3,338 (99.1%)	3,337 (99.1%)	3,336 (99.0%)	3,194 (94.7%)	3,221 (95.6%)
Manadotua (5 gaps)	3,352 (99.5%)	3,352 (99.5%)	3,350 (99.4%)	3,353 (99.5%)	3,353 (99.5%)	3,338 (99.1%)	3,337 (99.1%)	3,336 (99.0%)	3,194 (94.7%)	3,221 (95.6%)
Mantehage (5 gaps)	3,352 (99.5%)	3,352 (99.5%)	3,350 (99.4%)	3,353 (99.5%)	3,353 (99.5%)	3,338 (99.1%)	3,337 (99.1%)	3,336 (99.0%)	3,194 (94.7%)	3,221 (95.6%)
DUA (3 gaps)	3,351 (99.4%)	3,352 (99.4%)	3,350 (99.4%)	3,351 (99.4%)	3,351 (99.4%)	3,336 (99.0%)	3,335 (98.9%)	3,334 (98.9%)	3,192 (94.6%)	3,219 (95.4%)
LAB (5 gaps)	3,355 (99.6%)	3,354 (99.6%)	3,352 (99.5%)	3,354 (99.6%)	3,354 (99.6%)	3,339 (99.1%)	3,338 (99.1%)	3,337 (99.1%)	3,193 (94.7%)	3,220 (95.5%)
OGA (5 gaps)	3,352 (99.5%)	3,353 (99.5%)	3,351 (99.5%)	3,352 (99.5%)	3,352 (99.5%)	3,337 (99.1%)	3,336 (99.0%)	3,335 (99.0%)	3,191 (94.7%)	3,218 (95.5%)
BAT (3 gaps)	3,350 (99.4%)	3,351 (99.4%)	3,349 (99.3%)	3,350 (99.4%)	3,350 (99.4%)	3,335 (98.9%)	3,334 (98.9%)	3,333 (98.9%)	3,192 (94.6%)	3,219 (95.4%)
UWE (3 gaps)	3,353 (99.5%)	3,354 (99.5%)	3,352 (99.4%)	3,353 (99.5%)	3,353 (99.5%)	3,338 (99.0%)	3,337 (99.0%)	3,336 (99.0%)	3,195 (94.7%)	3,222 (95.5%)
KEN (4 gaps)	3,353 (99.5%)	3,354 (99.5%)	3,352 (99.5%)	3,353 (99.5%)	3,353 (99.5%)	3,338 (99.1%)	3,337 (99.0%)	3,336 (99.0%)	3,192 (94.7%)	3,219 (95.5%)
BAN (5 gaps)		3,359 (99.7%)	3,359 (99.7%)	3,361 (99.8%)	3,359 (99.7%)	3,348 (99.4%)	3,347 (99.3%)	3,346 (99.3%)	3,196 (94.8%)	3,223 (95.6%)
KAM (5 gaps)	3,359 (99.7%)		3,365 (99.9%)	3,363 (99.8%)	3,364 (99.9%)	3,342 (99.2%)	3,341 (99.2%)	3,340 (99.1%)	3,196 (94.8%)	3,221 (95.6%)
LAO (5 gaps)	3,359 (99.7%)	3,365 (99.9%)		3,362 (99.8%)	3,363 (99.8%)	3,341 (99.2%)	3,340 (99.1%)	3,339 (99.1%)	3,192 (94.7%)	3,219 (95.5%)

LUW (5 gaps)	3,361 (99.8%)	3,363 (99.8%)	3,362 (99.8%)		3,365 (99.9%)	3,346 (99.3%)	3,345 (99.3%)	3,344 (99.3%)	3,196 (94.8%)	3,223 (95.6%)
KOR (5 gaps)	3,359 (99.7%)	3,364 (99.9%)	3,363 (99.8%)	3,365 (99.9%)		3,345 (99.3%)	3,344 (99.3%)	3,343 (99.2%)	3,194 (94.7%)	3,221 (95.6%)
PEA (17 gaps)	3,348 (99.4%)	3,342 (99.2%)	3,341 (99.2%)	3,346 (99.3%)	3,345 (99.3%)		3,356 (99.97%)	3,355 (99.9%)	3,185 (94.5%)	3,213 (95.3%)
KOJ (17 gaps)	3,347 (99.3%)	3,341 (99.2%)	3,340 (99.1%)	3,345 (99.3%)	3,344 (99.3%)	3,356 (99.97%)		3,356 (99.97%)	3,184 (94.5%)	3,212 (95.3%)
MAK (17 gaps)	3,346 (99.3%)	3,340 (99.1%)	3,339 (99.1%)	3,344 (99.3%)	3,343 (99.2%)	3,355 (99.9%)	3,356 (99.97%)		3,184 (94.5%)	3,211 (95.3%)
Carlito (13 gaps)	3,196 (94.8%)	3,196 (94.8%)	3,192 (94.7%)	3,196 (94.8%)	3,194 (94.7%)	3,185 (94.5%)	3,184 (94.5%)	3,184 (94.5%)		3,260 (96.8%)
Cephalopachus (7 gaps)	3,223 (95.6%)	3,221 (95.6%)	3,219 (95.5%)	3,223 (95.6%)	3,221 (95.6%)	3,213 (95.3%)	3,211 (95.3%)	3,211 (95.3%)	3,260 (96.8%)	
